# Reverse Fosbury Flop Tear of the Rotator Cuff

**DOI:** 10.1155/2017/3635897

**Published:** 2017-09-05

**Authors:** Jérôme Tirefort, Gregory Cunningham, Alexandre Lädermann

**Affiliations:** ^1^Division of Orthopaedics and Trauma Surgery, La Tour Hospital, Geneva, Switzerland; ^2^Division of Orthopaedics and Trauma Surgery, Hirslanden Clinique La Colline, Geneva, Switzerland; ^3^Faculty of Medicine, University of Geneva, Geneva, Switzerland; ^4^Division of Orthopaedics and Trauma Surgery, Department of Surgery, Geneva University Hospitals, Geneva, Switzerland

## Abstract

**Introduction:**

“Fosbury flop” tear is an avulsion of the posterosuperior rotator cuff from the bone with reversal healing on its medial bursal-side. This case report describes a unique variant of Fosbury flop tear with a lesion of the musculotendinous junction that healed, for its tendon part, on the anterior humerus and coracoid process.

**Case Presentation:**

A 62-year-old man developed a posttraumatic painful shoulder with active loss of range of motion. Magnetic resonance arthrography demonstrated a lesion of the musculotendinous junction of the supraspinatus with healing of the tendon on the above-mentioned structures (reverse Fosbury flop). During arthroscopic evaluation, tendon repair was not possible and a debridement to avoid subacromial and anterior impingement associated with a tenotomy of the long head of the biceps were carried out. One year postoperatively, the patient had complete range of motion and was satisfied with the clinical results.

**Discussion and Conclusion:**

Different Fosbury flop tears exist. Radiologists and orthopedic surgeons should be aware of these tear patterns as failure to recognize them may lead to inadequate treatment.

## 1. Introduction

Massive rotator cuff tears comprise approximately 20% of all cuff tears and 80% of recurrent tears [1]. Among these massive tears, D type tears (full-thickness posterosuperior tears) according to Collin et al. [2] are the most common (35%) and come in a variety of patterns [3]. Amongst the latter, the Fosbury flop tear [4] (B3 in the Lädermann subclassification) [3] is a newly described lesion which occurs from a full-thickness tear that has flipped upon itself [4]. Failure to recognize this entity may lead to inadequate treatment with tendon mistakenly debrided as subacromial bursa or adhesions, resulting in a substantial loss of substance. We describe a unique variant of Fosbury flop tear with musculotendinous junction lesion that flopped and healed on the anterior humerus and coracoid process which we have termed the “reverse Fosbury flop” tear.

## 2. Case Presentation

**Figure 1:**
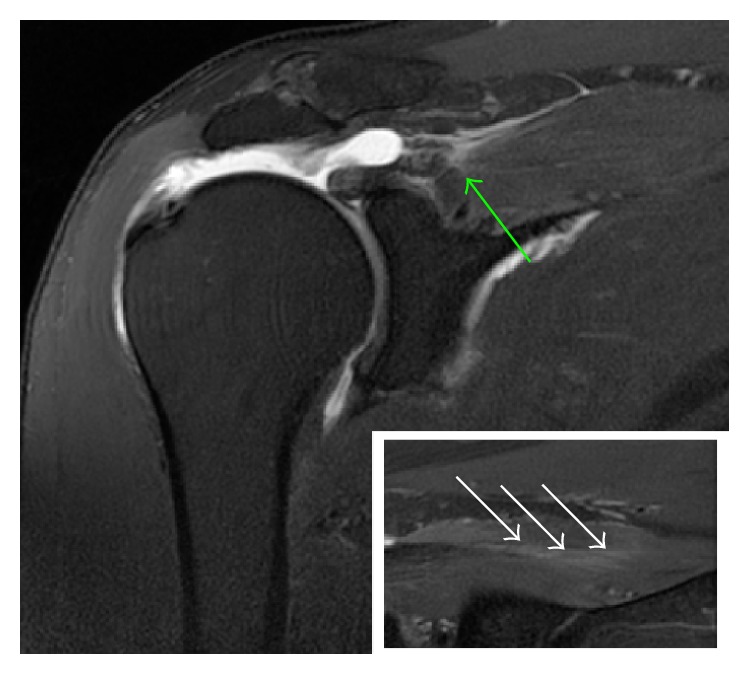
Coronal T2 weighted image with fat saturation of a right shoulder. A tear at the musculotendinous junction (green arrow) and a muscular edema (white arrows in the frame) are observed.

A healthy and athletic 62-year-old right-handed male with no previous history of shoulder pain sustained a direct fall on his right side during a skiing accident. He developed an immediate loss of active range of motion. A fracture was ruled out on standard radiographs, and the patient was referred two months later to a specialized shoulder clinic. On clinical examination, anterior active elevation was limited to 100 degrees, whereas passive motion was complete. Posterosuperior rotator cuff testing was deficient [5]. Magnetic resonance arthrography (MRA) revealed a complete lesion of the supraspinatus tendon at the musculotendinous junction (Figure 1) with associated muscular edema [6].

**Figure 2:**
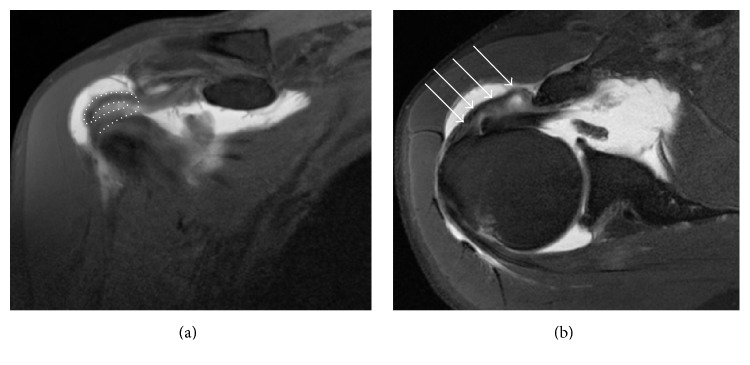
Coronal T1-weighted (a) and axial T2-weighted (b) with fat saturation of a right shoulder. The supraspinatus flopped on itself (white dotted line) and healed on the anterior humerus (white arrows).

The lesion did not fill the usual and undermentioned radiological Fosbury flop criteria [4] but had a unique pattern; the lesion corresponded to a musculotendinous junction lesion of the rotator cuff [6] that flopped on itself and healed on the anterior humerus and coracoid process (Figure 2).

**Figure 3:**
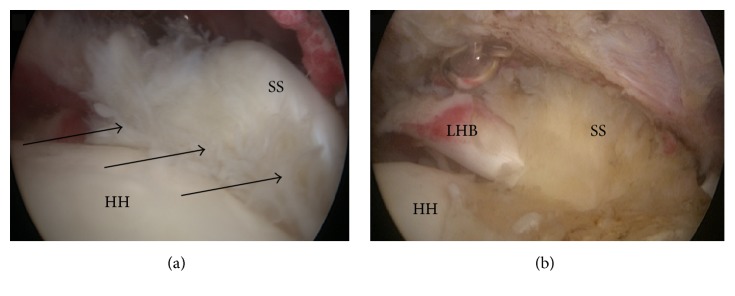
Arthroscopic view of a right shoulder viewed from posterior portal. (a) The supraspinatus tendon had the appearance of ulcerations and flanges of the bursal-side (sea anemone appearance, black arrows) and (b) after debridement of the flanges, the tendon that flopped on itself and the long head of the biceps and healed on the anterior humerus had an unusual orientation (HH, humeral head; LHB, long head of the biceps; SS, supraspinatus tendon).

The medial muscular stump had a grade 3 retraction according to Patte [7] and a grade 1 fatty infiltration according to Goutallier, supporting the traumatic nature of the lesion. An arthroscopy was performed three months after the trauma and confirmed the MRA aspect of the supraspinatus tendon lesion (Figure 3).

A release was done but unfortunately it was impossible to reattach the tendon on the muscle. Therefore, a simple debridement and a tenotomy of the long head of the biceps were performed. The patient was allowed to move his shoulder freely the day after the surgery. The patient recovered active motion in elevation three months after the surgery. At one-year follow-up, the pain decreased by 6 points from 8 points at baseline to 2 points on the pain visual analog scale, active anterior elevation improved to 150 degrees (versus 160 degrees on the other side), active external rotation was 50 degrees and symmetric, and internal rotation was slightly limited to L1 versus T10 for the other side. The constant [8] and the SSV [9] scores improved from 21 and 20 points preoperatively to 74 and 80 at one year, respectively.

## 3. Discussion

**Figure 4:**
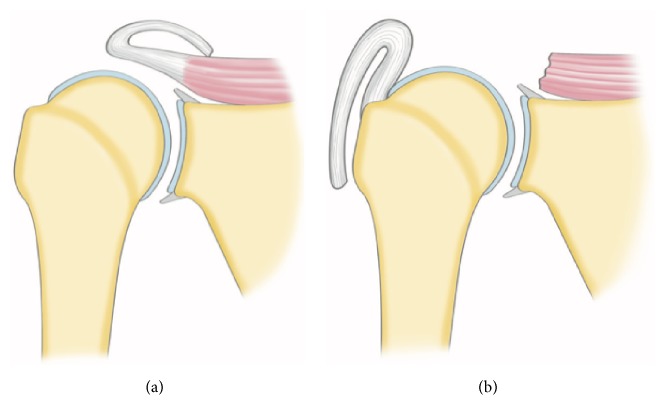
Illustration of a “Fosbury flop tear” (a) and of a “reverse Fosbury flop tear” (b).

Fosbury flop tear is an unusual avulsion of the posterosuperior cuff with reversal healing on its bursal-side. Five typical radiological signs have been described on MRA [4]: a thickened tendon (>9 mm), visualization of a tendon stump with a superomedial orientation, fluid accumulation in the superomedial part of the subacromial bursa, abnormal orientation of the fibers in the tendon stump on T1-weighted images with fat saturation, and adherence between the bursal tendon side and the wall of the subacromial bursa, with the two formers being the most sensitive isolated criteria for diagnosis (90.9%; CI: 62.3–98.4%; see [10]). In the present case, the tendon had a normal thickness of 7.2 mm. The tendon presented an anterolateral flop which turned on itself and healed on the anterior humerus and coracoid process; this is the reason why it was named “reverse Fosbury flop tear” (Figure 4).

This unusual pattern of healing was confirmed during arthroscopy (Figure 3). To the best of our knowledge and based on an advanced research on “PubMed,” this healing of the supraspinatus has never been described before. Indeed, with Fosbury flop tears representing only 2.6% of all posterosuperior rotator cuff tears [4] and musculotendinous junction lesions being also exceptional, the conjunction of these two pathologies explains the singularity of the presented case. The clinical implication of this finding is, first and foremost, that a good anatomic understanding of the rotator cuff tear is crucial to allow an anatomic repair and proper tendon stump identification intraoperatively. Although this particular lesion was not repairable because of its musculotendinous failure pattern, knowledge that this variation of Fosbury flop tear can occur and identifying it on preoperative imaging may avoid extensive damage to the tendon when carrying out a routine rotator interval and subcoracoid clearing during the first intra-articular step. Secondly, although repair was not possible in this case, the patient still presented a good outcome, with a near complete recovery of range of motion and a good quality of life, at one year postoperatively. This corroborates the known fact that massive posterosuperior rotator cuff tears (D type of Collin) [2] with an intact subscapularis have a good prognosis after conservative treatment [11].
